# The Peculiar Trialogue between Pediatric Obesity, Systemic Inflammatory Status, and Immunity

**DOI:** 10.3390/biology10060512

**Published:** 2021-06-09

**Authors:** Lorena Elena Meliț, Cristina Oana Mărginean, Cristian Dan Mărginean, Maria Oana Săsăran

**Affiliations:** 1 Department of Pediatrics I, George Emil Palade University of Medicine, Pharmacy, Science and Technology of Târgu Mureș, Gheorghe Marinescu Street No 38, 540136 Târgu Mureș, Romania; lory_chimista89@yahoo.com (L.E.M.); marginean.cristiandan@gmail.com (C.D.M.); 2 Department of Pediatrics III, George Emil Palade University of Medicine, Pharmacy, Science and Technology of Târgu Mureș, Gheorghe Marinescu Street No 38, 540136 Târgu Mureș, Romania; oanam93@yahoo.com

**Keywords:** pediatric obesity, systemic inflammatory status, immunity

## Abstract

**Simple Summary:**

The dogma that adipose tissue is a simple energy storage tissue is no longer accepted since it has been proved that it also has an incontestable multifunctional role acting like a true standalone organ resembling to endocrine or immune organs. Nevertheless, the scarcity of longitudinal studies involving pediatric subjects hinder disclosure regarding the long-term effect of this inflammation into adulthood and the outcome of interventional strategies on reducing the complications associated with this low-grade systemic inflammation.

**Abstract:**

Pediatric obesity is not only an energetic imbalance, but also a chronic complex multisystem disorder that might impair both the life length and quality. Its pandemic status should increase worldwide awareness regarding the long-term life-threatening associated complications. Obesity related complications, such as cardiovascular, metabolic, or hepatic ones, affect both short and long-term wellbeing, and they do not spare pediatric subjects, defined as life-threatening consequences of the systemic inflammatory status triggered by the adipose tissue. The energetic imbalance of obesity clearly results in adipocytes hypertrophy and hyperplasia expressing different degrees of chronic inflammation. Adipose tissue might be considered an immune organ due to its rich content in a complex array of immune cells, among which the formerly mentioned macrophages, neutrophils, mast cells, but also eosinophils along with T and B cells, acting together to maintain the tissue homeostasis in normal weight individuals. Adipokines belong to the class of innate immunity humoral effectors, and they play a crucial role in amplifying the immune responses with a subsequent trigger effect on leukocyte activation. The usefulness of complete cellular blood count parameters, such as leukocytes, lymphocytes, neutrophils, erythrocytes, and platelets as predictors of obesity-triggered inflammation, was also proved in pediatric patients with overweight or obesity. The dogma that adipose tissue is a simple energy storage tissue is no longer accepted since it has been proved that it also has an incontestable multifunctional role acting like a true standalone organ resembling to endocrine or immune organs.

## 1. Pediatric Obesity–A Worldwide Public Health Problem

Pediatric obesity has become a current challenging trend in pediatrics. Multiple studies focused on assessing this multisystem nutritional disorder due to its alarmingly high incidence being therefore considered a worldwide public health problem with a negative impact on both individual’s life quality and national health services. Thus, according to the most recent reports of the World Health Organization, pediatric obesity affects approximately 40 million children aged below five years, pointing out a higher prevalence in younger children [1]. Moreover, age and socioeconomical level were not found to influence the increasing incidence of this condition [1]. It is well known that every child with obesity carries a higher risk of becoming an adult with obesity, suggesting the imminent long-term persistence of this condition. Genetic susceptibility is clearly a solid background for the development of obesity being proved that this condition originates even from the intrauterine life and that children whose mothers carry a susceptible genetic profile are more prone to have a higher birth weight and to experience an excessive weight gain during childhood [2,3]. In spite of the essential role of genetic profile, obesity occurs only in the setting of the so-called ‘obesogenic factors’ suggesting that environment plays a dichotomous role in the etiology of this multifactorial disorder since it is the single modifiable factor [4].

Obesity related complications, such as cardiovascular, metabolic, or hepatic ones, defined as life-threatening consequences of the systemic inflammatory status triggered by the adipose tissue, affect both short and long-term wellbeing and they do not spare pediatric subjects [5,6,7]. Thus, atherosclerosis was proven to occur during childhood in the setting of obesity and the awareness of the early onset of this major cardiovascular risk factor, commonly overlooked at young ages, might hinder its persistence into adulthood [8,9]. A recent study of our team emphasized the alteration of lipid profile parameters in children with overweight and obesity underlining that this group presented significantly higher levels of total cholesterol, LDL-cholesterol, and triglycerides associated with a lower HDL-cholesterol in comparison to normal weight children [7]. Taking into account that the association between increased LDL-cholesterol and low HDL-cholesterol is mandatory for the formation of atherosclerotic plaques [10], the previously mentioned study sustains that cardiovascular risk associated with obesity is not related to the patient’s age and imminently worsens in time. Metabolic complications are fairly related to the impairment of lipid profile in patients with obesity since dyslipidemia, i.e., the association between increased triglycerides and low levels of HDL-cholesterol, was identified as a potential trigger of insulin resistance [11]. A lipid panel might also be useful in diagnosing fatty liver disease in patients expressing metabolic features, combined with other non-invasive tests, such as liver transaminases, total and direct bilirubin, insulin level, and fasting glucose [12]. Liver impairment associated with obesity was also described in children afflicted by this condition being reported that non-alcoholic fatty liver disease (NAFLD) is the most common form of pediatric chronic liver disease, with a prevalence between 5–17% in Western countries [13]. It is a well-known fact that liver has the ability to regenerate and that hepatic steatosis is a reversible process if its trigger is no longer present. Nevertheless, due to the lack of appropriate management, liver steatosis might turn into fibrosis and eventually into cirrhosis, which is no longer reversible. The assessment of liver impairment in patients with obesity is particularly important in order to prevent the irreversible cirrhotic transformation of the hepatocytes and to increase the patient’s or family’s awareness in the case of children regarding the importance of weight loss. The fluctuations of aminotransferases alter their usefulness in diagnosing fatty liver disease since it was proven that children with obesity associated with NAFLD or non-alcoholic steatohepatitis (NASH) might have normal levels of aspartate aminotransferase (AST) or alanine aminotransferase (ALT) over time [14]. Moreover, NALFD was proven to be present in up to 9.6% of overweight children and almost 50% of those with obesity [13,15,16] underlining a current emergent need for a more reliable non-invasive test to establish this diagnosis. Although liver biopsy remains the gold standard for detecting NAFLD or NASH, its utility is limited in pediatric patients due to the potential complications related to its invasiveness and the subsequent parent’s poor compliance. Elastography methods have recently emerged to fulfill this crucial need, but the data on children are rather scarce and far from being clarified. Both transient and 2D shear wave elastography methods were identified as reliable quantitative biomarkers in the assessment of liver stiffness associated with pediatric obesity [17,18,19]. The diagnostic accuracy of these methods could be augmented by combining the elastography parameters with aminotransferase levels or other indices, such as the aspartate aminotransferase/platelets ratio index or the AST/ALT ratio [19].

The persistent increase in incidence along with its short and long-term life-threatening complications provides obesity with a leading position among the diseases defined as public health problems worldwide. The peculiarities of pediatric obesity result mainly from the urgent need of the family/care-givers to actively participate in the management of this condition. Therefore, communication between physician, family, and children represents a cornerstone in building an effective partnership for the patient’s best outcome [20].

## 2. The ‘Give and Take’ between Immunity and Systemic Inflammation Associated with Pediatric Obesity

Chronic systemic inflammation associated with pediatric obesity is no longer a red flag among researchers. Thus, adipose tissue has been defined as peculiar due to its involvement in both the initiation and maintenance of this unfortunate association between obesity and low-grade systemic inflammation [5,21]. The deficiency of immune activity related to adipocytes is the main cause of the above-mentioned inflammation, and it implies neutrophil participation in the early stages with their temporary infiltration within the abdominal fat [21,22,23]. The inflammatory cascade continues by mast cell polarization and macrophage attraction to actively participate in this inflammatory process, it being disclosed that, along with pre-adipocytes, they are involved in the production of cytokines [22,23,24]. Therefore, the energetic imbalance of obesity clearly results in adipocyte hypertrophy and hyperplasia expressing different degrees of chronic inflammation at this level and further regulating the occurrence of certain chronic conditions also known as obesity-related complications [24].

There has been an explosion regarding the cause–effect link between immune cells and inflammation induced by adipose tissue and its consequences on the development of obesity-associated wide-spectrum complications, such as cardiovascular diseases related to dyslipidemia, NAFLD, and NASH or metabolic syndrome including insulin resistance and subsequent type 2 diabetes mellitus, all of them being reported with a recent increasing incidence in children with obesity. Taking into account the well-documented trialogue between obesity, inflammation, and the above-mentioned conditions in adults, we might hypothesize that pediatric patients follow the same pattern. Therefore, adipose tissue might also be considered an immune organ due to its rich content in a complex array of immune cells, among which the formerly mentioned macrophages, neutrophils, mast cells, but also eosinophils along with T and B cells, acting together to maintain the tissue homeostasis in normal weight individuals [25,26].

It was emphasized that the accumulation of macrophages within the visceral abdominal fat should be considered a key pathogenic feature of obesity since visceral adiposity accurately predicts fatty liver disease, cardiovascular conditions, and type 2 diabetes mellitus in comparison with subcutaneous fat, which poses a lower or no risk at all in these conditions [27,28]. Moreover, studies on obese individuals proved that macrophages tend to accumulate in a greater amount in the visceral omental fat as compared to subcutaneous inguinal fat depot [29]. Taking into account that macrophages also exist in the lean tissue, we might hypothesize that these immune cells have a dichotomous role expressing both protective and damaging functions in the metabolic regulation process [30]. Th1 (M1) and Th2 (M2) polarizing signals have been postulated as potential explanations for the differential responses to macrophages being proved that obesity-associated inflammation results in an increase of M1 gene markers and a decrease of M2 ones in adipose tissue [31,32,33]. It was also proved that adipose tissue owns the ability to promote infiltration and migration of macrophages, being capable of inducing a shift in macrophage production towards the M1 phenotype [34]. Therefore, a study performed on mice underlined that obesity reduces the M2:M1 ratio from 4:1 in normal weight mice to 1.2:1 to 1.2:1 in those overfed [35]. Nevertheless, this process seems to be reversible as it was shown in two studies performed on mice, which were switched from a high-fat diet to a chow diet or treated with thiazolidinediones [36,37] revealing a switch in adipose tissue macrophages polarization from M1 to M2 and a subsequent improvement in insulin sensitivity [28].

As we already mentioned, mast cells are also involved in obesity-induced inflammation. The relationship between mast cells and inflammation is undoubtable since activated mast cells were proved to secrete a wide spectrum of inflammatory mediators such as histamine, heparin, proteases, lipid mediators, as well as pro- and anti-inflammatory cytokines (TNFa, IL1b, IL6, TGFb, IL4, and IL10) [38]. Moreover, it was hypothesized that mast cells might be involved in adipose tissue regulation through angiogenesis since the deficit of these type of immune cells was associated with diminished angiogenesis [39]. The distribution of this type of immune cells differs in lean mice when compared to those with obesity. Thus, Altintas et al. showed that, in the setting of obesity, mast cell density significantly increases in visceral fat in comparison with subcutaneous fat where no substantial changes were noticed [27]. Additionally, Liu et al. noticed that mice fed on a Western diet, in which mast cells suffered either a genetically induced deficiency or a pharmacological stabilization, presented a reduction in both body weight gain and adipokine levels [39].

Eosinophils, another cellular subtype, are active participants in anti-inflammatory immune responses due to their secretion of Th2 cytokines, among which IL4, IL10, IL13, or TGFβ, being also involved in macrophage M2 polarization and Th2 differentiation [40]. Their involvement in the development of adipose tissue inflammation was suggested by Wu et al., who noticed that mice on a high-fat diet with genetically induced eosinophils deficiency present an increase in body weight along with insulin resistance and impairment of glucose tolerance [41]. Moreover, IL4, an insulin-sensitizing factor, was proven to be mainly secreted by adipose tissue-resident eosinophils [41].

Based on these findings, the authors stated that eosinophils might be defined as the central driver of M2 differentiation. Furthermore, a reciprocal relation was underlined between adipose tissue eosinophils and innate lymphoid type 2 cells via IL5 and IL13 [42]. Therefore, it is still unclear whether eosinophils directly influence obesity-associated insulin resistance and adipose tissue-related inflammation or if these are a result of eosinophils effects on body weight and adiposity changes [43].

Lymphocytes and neutrophils are not spared by the complex inflammatory process identified in individuals with obesity. It was stated that neutrophils are a better indicator of the inflammatory status being directly related to obesity degree, whereas lymphocytes better reflect the nutritional status and general stress [44,45]. T lymphocytes are able to influence the adipose tissue macrophage count and to affect their polarization. Thus, T-helper (Th) cells comprise Th1 subset known to produce pro-inflammatory cytokines and Th2 subset with a contrary function, i.e., production of anti-inflammatory cytokines [28]. CD4+ Th1 cell density is positively correlated with NAFLD, and they mainly secrete IFNγ, which was proved to promote macrophages M1 polarization [43]. Moreover, IFNγ seems to favor insulin resistance independently of body weight changes since improved insulin resistance and diminished adipose tissue inflammation were noticed in IFNγ deficient mice [46]. On the contrary, according to the findings of Winer, no improvement of insulin resistance or obesity-associated inflammation was found in mice with impaired Th2 development, but normal development of Th1 cells [47]. Thus, Th2 lymphocytes act as suppressive factors for both adipose tissue inflammation and insulin resistance and most-likely the shift in Th1/Th2 ratio favoring Th1 proinflammatory subset results in a subsequent shift in polarization from M2 to M1 [43].

Another type of T cells, regulatory T cells (Treg), express their benefic function by inhibiting macrophage migration and inducing M2 polarization [48]. Furthermore, Treg showed a substantial reduction in genetically obese mice or those fed on a high-fat diet, with a subsequent increase in visceral abdominal fat adipokine levels [48]. Feurer et al. proved that Treg cells depletion into adipose tissue results in increased insulin resistance promoting local and systemic production of proinflammatory cytokines, while exogenous stimulation of Treg subset via IL12 is associated with improved insulin resistance and increased IL10 levels [48]. Increased glucose uptake into adipocytes, hindrance of Th1 differentiation at the level of adipose tissue, as well as diminished M1 polarization, were hypothesized as possible mechanisms for amelioration of insulin resistance and obesity-associated inflammation via Treg cells [48,49]. Adipose tissue Treg cells are peculiar due to their ability to produce PPARγ, a well-documented regulator of adipocyte differentiation. Moreover, a study performed on mice proved that Treg-specific deletion of PPARγ resulted in a decrease of Treg cells only in adipose tissue [50].

The relationship between Th17 and Treg cells has been outlined as a cornerstone in terms of metabolic dysfunction pathogenesis. It is well-documented that these two subpopulations of T lymphocytes have opposite effects and reciprocal developmental pathways since Th17 acts as pro-inflammatory cells while Treg display regulatory functions [30,31]. Therefore, the imbalance between Treg/Th17 as it occurs in patients with obesity will result in the development of obesity-associated complications, such as insulin resistance and subsequent type 2 diabetes mellitus, NAFLD or atherosclerosis [51,52,53,54,55,56], which were also reported in young age groups [7,19,57,58,59]. Thus, the involvement of lymphocyte subpopulations imbalance in the pathogenic mechanisms of systemic inflammation and dysmetabolism is incontestable, but its proper elucidation in pediatric population is far to be achieved. Nevertheless, Calcaterra et al. performed a study on obese versus normal weight children and proved that Th17 and Treg distribution is significantly correlated to the presence of insulin resistance and hypertension [60]. These findings not only support the hypothesis of T subpopulations imbalance influence on systemic inflammation, but also suggest that the aforementioned conditions are interrelated and that low-grade systemic inflammation associated with obesity is involved in early angiogenesis [51,56,60]. The proper identification of T cells influence in protection or predisposition to obesity related complication is crucial in pediatric populations since the implementation of effective preventive strategies at young ages would definitely decrease the health and economic burden during adulthood.

The hypothesis of adaptive immunity involvement in obesity-induced inflammation is further sustained by the expansion of a visceral abdominal fat B-cell population in mice fed on a high-fat diet with a subsequent increase in tissue immunoglobulin M and G levels [61]. Moreover, it is a well-documented fact that adipose tissue expresses an increased number of B cells, and a depletion of these lymphocytes leads to an improvement in insulin resistance [61,62]. Studies performed on obese animals showed that obesity leads to increased levels of B-cell derived IgG2c antibodies. Moreover, the transfer of these antibodies into lean mice triggered adipose tissue specific inflammation and subsequent development of insulin resistance [61]. The same study proved that this reaction was possible only in the presence of T cells emphasizing the incontestable interdependence between B and T lymphocytes. It was further hypothesized that B-cell derived IgG2c antibodies have the ability to shift macrophage polarization from M2 to M1 phenotype since B-cell deficient mice express a reduction of M1 polarization [61]. Moreover, these antibodies were proven in vitro to induce the macrophage production of TNFα [61].

Neutrophils transiently infiltrate the visceral abdominal fat and bind to the adipocytes preceding macrophage infiltration at this level [63]. This transient pattern of infiltration was suggested by Elgazar-Carmon et al., who noticed a decrease in adipose tissue neutrophils after one week of high-fat diet in spite of their initial outstanding increase [21]. Thus, neutrophils are able to induce the recruitment and activation of the second-line immune cells among which macrophages, dendritic cells, and lymphocytes due to their increased secretion of cytokines and chemokines (TNFa, IL1b, IL8, and CCL3) [63,64]. Moreover, neutrophils might be considered as connectors between innate and adaptive immunity components [65]. In spite of their low number within adipose tissue in normal settings, they migrate at this level even after short-term exposure to high-fat diet [21]. This leukocyte subtype is also known as the most important and abundant one in the human peripheral blood with a significant increase in individuals with obesity [6,66]. Moreover, neutrophil count was proven to correlate with total adipose tissue, waist circumference, and body mass index in obese female teenagers [67]. On the contrary, studies performed on younger children failed in identifying this trend in smaller children with obesity, most-likely due to the insufficient amount of time required for this process to worsen enough taking into account that neutrophils reflect obesity degree [6]. The increased levels of plasma MPO, neutrophil activation marker CD66b, and calprotectin, mainly originating from neutrophils, in individuals with obesity as compared to lean controls sustain the relationship between obesity and systemic activation of neutrophils [68]. Neutrophils are considered the earliest immune cells recruited into adipose tissue based on the findings of Ferrante et al., who found an increase of 20-fold in adipose tissue neutrophils within the first three days after the initiation of high-fat diet, while the increase in macrophages was noticed only after seven days [69]. Therefore, we might state that neutrophils own a pivotal role in the initiation of adipose tissue related inflammation.

Dendritic cells represent probably the least studied type of immune cells in terms of adipose tissue inflammation. Nevertheless, it was proved that dendritic cells secrete a broad spectrum of cytokines that influence adaptive immunity maturation and activation, among which IL12 involved in the differentiation of Th1 from naïve T lymphocytes and IL15, which promote the proliferation of CD8+ T lymphocytes [70]. Thus, it was proved that a high-fat diet results in an increase in adipose tissue-specific dendritic cells and that the subcutaneous tissue of obese individuals display elevated expression of dendritic cells antigens in comparison with lean controls [71]. Additionally, induced genetic ablation of dendritic cells led to a decrease in adipose tissue macrophages, being associated with improved insulin resistance, effects that might be related either to dendritic cell deletion or to the weight loss induced by the mutation [72].

The roles of different types of cells involved in regulations of the bridge among obesity, immunity, and systemic inflammation are related in Table 1.

Adipokines belong to the class of innate immunity humoral effectors, and they play a crucial role in amplifying the immune responses with a subsequent trigger effect on leukocyte activation [30]. According to one of the previously mentioned studies, a decrease in adipose tissue-induced pro-inflammatory cytokines, i.e., adipokines, was noticed in mice as a result of switching from a high-fat diet to a chow diet [36]. Certain cytokines, such as tumor necrosis factor (TNF), interleukin (IL) 1 or interferon, seem to contribute to the relationship between obesity-associated inflammation and insulin resistance since disruption of these signals resulting in improved glucose homeostasis in mouse models [74]. Therefore, incontestably, adipose tissue expresses a secretion function consisting of both hormones (leptin, resistine, adiponectin) and cytokines (IL 1, 6, TNF α, etc.) [70]. The paracrine and endocrine functions of the adipose tissue are further sustained by the synthesis of other molecules like bioactive lipids and RNA molecules, whose production depends on the energy status of the adipose tissue [75]. Leptin and adiponectin are two adipose-tissue specific hormones, the first one being involved in appetite suppression, energy expenditure promotion, heat loss regulation [76], and insulin resistance [77,78], while the second acts as a systemic insulin-sensitizing and anti-inflammatory systemic factor [79]. Adipsin or complement factor D is another adipose tissue-derived molecule displaying a dichotomous role depending on disease severity, proinflammatory via the production of chemotactic molecules, or anti-inflammatory through opsonization and removal of dead cells [75]. Fatty acid-binding protein 4 (FABP4) and neuregulin 4 are another two proteins secreted by adipocytes with relatively opposite functions, since FABP4 was associated with obesity, insulin resistance, type 2 diabetes mellitus, and cardiovascular risk [80], while neuregulin 4 seems to counteract obesity-associated complications like chronic inflammation and metabolic changes in the liver [75,81]. As we already mentioned, adipose tissue was proved to also secrete lipokines, among which palmitoleate with insulin-sensitizing and anti-inflammatory effects [82,83], fatty acid esters of hydroxy fatty acids that might act as endocrine and antidiabetic regulators [75], and 12,13-dihydroxy-(9Z)-octadecenoic acid with paracrine and endocrine functions increasing fatty acid uptake in brown adipose tissue and skeletal muscles [84,85]. Moreover, the obesity-resident immune cells were also proved to secrete certain adipokines like: C-X-C motif chemokine ligand 14, released from brown adipose tissue, seems to be involved in the stimulation of thermogenic differentiation in white adipose tissue via M2 macrophage polarization [75]; IL 6 which might express both a proinflammatory and anti-inflammatory effects depending on cell type and context being involved in hepatic glucose metabolism and insulin resistance [75,86]; resistin, a protein secreted by both adipocytes and macrophages, which promotes insulin resistance and the development of metabolic disorders by acting on adipocytes, hepatocytes, macrophages, myocytes, endothelial cells, and hypothalamic neurons [87]; nicotinamide phosphoribosyltransferase or visfatin, a protein associated with obesity and insulin resistance with a proved contribution in atherosclerosis and diabetes pathogenesis [88,89]; omentin or intelectin 1, a protein highly-expressed in omental white adipose tissue, whose low plasma levels were associated with insulin resistance [90]; and vaspin or serpin A12, an insulin-sensitizing protease inhibitor [88]. Therefore, we might definitely support the statement that, in obese individuals, hypertrophic adipocytes and adipose tissue-resident immune cells augment the chronic, pro-inflammatory status via an altered secretion of adipokines and lipokines with subsequent increased cardiometabolic risk [75]. Moreover, these molecules express an endocrine action regulating glucose and lipid metabolism, appetite, and thermogenesis [75].

In terms of daily clinical practice, it was stated that increased IL6, IL1 β, and C-reactive protein (CRP) serum levels might precede the onset of type 2 diabetes mellitus, serving as accurate predictors for this condition [91]. Except for type 2 diabetes mellitus, adipokines are also involved in the pathogenesis of other obesity-associated complications, which include metabolic syndrome or NAFLD [92,93]. Pediatric populations with obesity tend to follow a similar pattern since a recent study underlined that leptin, IL6, and TNF α serum levels significantly correlate to body mass index in children with obesity [7]. Taking into account the role of IL1 β, IL6, and TNF α as central mediators of inflammatory reactions and their essential interaction in the synthesis of acute-phase reactants, we emphasize once more the role of peripheral blood markers in detecting the subclinical inflammation associated with obesity [7]. Moreover, neutrophils, also known as the prime effectors of inflammatory responses, have the ability to secrete TNF α and IL1 β [73].

The IL1 family of ligands comprises IL1α, IL1β, and IL1Ra. IL1α and IL1β are mainly synthetized by macrophages, but also by monocytes, neutrophils, or hepatocytes [94]. IL1α is the least studied of all three Il1R ligands in terms of obesity. Nevertheless, studies on mice proved that it might be involved in inhibiting adipocyte differentiation and diminishing insulin signaling [95]. Moreover, elevated levels of serum IL1α were found not only in obese mice [95], but also humans with obesity [96]. IL1β, one of the most abundant cytokine in plasma, along with IL1Ra are increased in the visceral adipose tissue [94]. As it was previously stated, visceral adipose tissue owns an essential role in the secretion of cytokines and development of insulin resistance in the setting of obesity [94]. A close link between IL1β and NLRP3 inflammasome has been identified by Dixit group who found that mRNA levels of both IL1β and NLRP3 were positively correlated to body weight and adiposity in high-fat diet-fed mice. Moreover, a significant reduction of these mRNAs was noticed after a caloric restriction diet [97]. These findings were confirmed in abdominal subcutaneous adipose tissue biopsies from males with obesity and type 2 diabetes mellitus, as well as subjects on a weight loss program [97]. Moreover, induced deficiency of NLRP3 or IL1β presented protective effects against developing inflammation and insulin resistance in the setting of a high-fat diet [98]. The complex interrelationship between all facts mentioned above is once more emphasized by the major role of macrophages in producing NLRP3 inflammasomes within adipose tissue [99]. Taking into account that adipose tissue inflammation is defined as an innate immune response, the toll-like receptor 4 (TLR4) was proven to have a crucial role in initiating the changes required for the transformation of healthy lean adipose tissue into inflammatory adipose tissue [100]. This role is sustained by the involvement of TLR4 in the formation of NLRP3 inflammasome [94].

IL33, another member of the IL1 cytokine family, was proved to play a protective role in the setting of obesity and type 2 diabetes mellitus [101]. Therefore, significantly increased serum levels of IL33 were found in the adipose tissue of patients with severe obesity [102]. Moreover, the IL-122Rα receptor of IL33 (or ST2) was also found to be overexpressed by the endothelial cells within the adipose tissue [103]. A close interaction between IL33 and Treg cells was revealed by Vasanthakumar et al., who pointed out that Treg cells within visceral adipose tissue express increased levels of ST2 and are reduced in mice with induced IL33 deficiency [104]. Another important interdependence between innate and adaptive immunity in the setting of obesity-associated inflammation was underlined by Brestoff et al., who found that IL33 might act as a crucial regulator of adipose tissue via group 2 innate lymphoid cells [105]. A study performed on cultured pre- and mature adipocytes pointed out a relationship between IL33 and TNF α finding an increase in IL33 expression as a consequence of TNF α stimulation [106]. Moreover, IL33 was considered a modulator of adipose tissue metabolism [107], it being proved that IL33 administration in mice results in a reduction in both body weight and fat mass [101,104,105]. Thus, it was proved that IL33 treatment is associated with adipocyte mean size, and that this interleukin downregulates the expression of adipogenesis genes inhibiting fat storage as it was shown in vitro during adipocyte differentiation [101,104]. Additionally, the administration of IL33 in obese mice was associated with the restauration of Treg cells proportion within visceral adipose tissue, reducing the number of macrophages infiltrating the adipose tissue, which further resulted in a lower expression of TNF α mRNA and increased insulin sensitivity [104,108]. Similar findings were outlined in humans by Hasan et al., who identified a negative correlation between IL33 and BMI, proving that overweight subjects have low serum levels of IL33 [102]. Indeed, IL33 is a strong modulator of adipose tissue homeostasis in both physiological and pathological settings, such as obesity.

IL18 is a key factor in metabolic homeostasis [109]. Several studies proved that high levels of this interleukin are associated with obesity [110], which significantly decrease as a result of massive weight loss after bariatric surgery [109] or diet-induced weight loss programs [111]. On the contrary, studies performed on IL18-deficient mice proved that these developed late onset obesity and insulin resistance reversible after IL18 administration [112,113]. Moreover, the production of IL18 seems to be related to NLRP1 inflammasome in the setting of metabolic/caloric stress [114]. The local effects of IL18 within adipose tissue are worth mentioning since they are essential contributors of adiposity [94].

IL6 is a peculiar proinflammatory adipokine defined as ‘metabolic hormone’, which influences glucose, lipid, and protein metabolism [115]. It was proved that this interleukin owns an essential role in blocking cell apoptosis favoring their survival during inflammation [116]. Taking into account that IL6 was found to be elevated in obese individuals, but also in the setting of type 2 diabetes mellitus and insulin resistance [117], we might emphasize that this interleukin could represent a ‘bridge’ between obesity and obesity-related systemic complications. Multiple gene polymorphisms of IL6 were studies in terms of obesity risk. Thus, a recent review revealed that the minor allele A of IL-6 rs1800797 gene polymorphism has a protective role against obesity, while the C allele of IL-6 rs1800795 gene polymorphism is associated with increased obesity risk [117]. Moreover, the interaction between IL6 and IL1 β is essential for the synthesis of C-reactive protein, an important player in systemic inflammation [6]. It seems that, within adipose tissue, IL6 is secreted by pre-adipocytes, which are small nucleated cells intermediating the transformation of mesenchymal cells into mature adipocytes [74,118]. Based on all the aforementioned facts, it is incontestable that adipose tissue is able to trigger and maintain a systemic inflammation via IL6, a hallmark of proinflammatory cytokines. Nevertheless, IL6 might express also an anti-inflammatory activity mediated by classic signaling mode, while the proinflammatory role identified in the setting of obesity is mediated by the trans-signaling pattern [119]. These findings might represent a solid basis for the development of effective weight loss innovative strategies if the factors involved in triggering each signaling mode are clearly identified. The role of cytokines produced by adipose tissue is described in Table 2.

MicroRNAs (miRNAs) are short noncoding RNA molecules that seem to own an essential role in adipogenesis and regulation of metabolic and endocrine functions of adipocytes [120]. Taking into account that both hyperplasia and hypertrophic expansion are essential for adipogenesis, multiple studies focused on identifying the factors that promote or suppress these processes. Thus, studies that compared miRNA expression in obese and lean white adipose tissue underlined that only 35 of the 574 identified miRNAs are differently expressed in these tissues [120]. MiRNA 221 is among the few miRNAs that was consistently found to be differentially expressed in the white adipose tissue of obese patients as compared to that of lean individuals, but the results remain controversial since studies concluded that it might be either upregulated or downregulated [120]. Moreover, miRNA 221 was associated with both the synthesis of TNF and the development of insulin resistance [121,122]. Therefore, it is undoubtable that these miRNAs also contribute to the development of obesity-related complications. Another fact worth mentioning is the distribution of these miRNAs between subcutaneous and omental white adipose tissue which seems to influence the development of obesity-related complications. Thus, it was proved that, despite the similarities of miRNAs expression in these depots, omental fat owns a more important role in terms of gene expression associated with type 2 diabetes mellitus as compared to subcutaneous adipocytes [120]. Another recent study supporting the previously reported findings concluded that the overexpression of miRNA 24, miRNA 30d, and miRNA 146a within abdominal adipose tissue might also be associated with both obesity and type 2 diabetes mellitus [123]. On the contrary, another recent study revealed that miRNA 146a might be involved in the regulation of adipocyte-related inflammation contributing to the prevention of systemic inflammation development triggered by obesity [124]. Except for miRNA 146a, other miRNAs, such as miRNA 132, 223, 221, or 146b were also found to be involved either in the promotion or suppression of obesity-associated inflammation [120]. Furthermore, the importance of WAT-specific miRNA regulatory networks was recently underlined, it being proved that they act as regulatory factors in obesity-associated inflammation [120]. Thus, miRNAs target different adipogenesis-regulating pathways resulting in a strong influence on adipogenesis like in the case of miRNA 30c, which acts as a promoter of adipocyte differentiation by targeting directly the interconnected transcripts, SERPINE and ACVR1 [125]. It was also proved that, in the setting of type 2 diabetes mellitus, most miRNAs act in a complementary manner increasing reciprocally their effects [120]. This synergistic pattern was also found in Th2 cells, where miRNA 223 and 139-3p complementary regulate IL5 and IL13 [126]. Given all these complex direct and indirect interrelations between miRNA networks, resulting in a strong control on human white adipose tissue inflammation and subsequent amplification of miRNAs effects on the metabolic and endocrine functions of adipocytes, they might represent a valuable basis for the development of innovative therapies targeting obesity-induced complications. Nevertheless, further studies, especially on pediatric populations, are required in order to define the precise role of miRNAs and their networks in pediatric obesity.

The usefulness of complete cellular blood count parameters, such as leukocytes, lymphocytes, erythrocytes, and platelets as predictors of obesity-triggered inflammation, was also proved in pediatric patients being overweight or obese [6,127]. Furthermore, certain studies indicated an association between white blood cells and the development of metabolic syndrome [128,129], emphasizing once more the role of obesity-associated inflammation as a mediator of all complications that might occur as a result of this pandemic condition.

Based on all the aforementioned data, we might definitely state that obesity-induced inflammation is a bona fide feature in children with obesity. Thus, this inflammatory status is not only initiated during childhood, but it worsens fast enough to also trigger the development of associated complications taking into account the current increasing incidence trend of NAFLD, type 2 diabetes mellitus, metabolic syndrome, or other obesity-induced burdens in pediatric patients worldwide.

## 3. Conclusions

The dogma that adipose tissue is a simple energy storage tissue is no longer accepted since it has been proved that it also has an incontestable multifunctional role acting like a true standalone organ resembling endocrine or immune organs. Pediatric obese patients are not spared by the life-threatening consequences of the systemic inflammatory status triggered by the adipose tissue such as atherosclerosis, NAFLD, NASH, cirrhosis, insulin resistance, and type 2 diabetes mellitus, which definitely alter the short- and long-term wellbeing of these individuals. Nevertheless, the scarcity of longitudinal studies involving pediatric subjects hinders disclosure regarding the long-term effect of this inflammation into adulthood and the outcome of interventional strategies on reducing the complications associated with this low-grade systemic inflammation. Therefore, the question mark stands for the point beyond which the systemic inflammation-associated with pediatric obesity is no longer reversible.

## Figures and Tables

**Table 1:** The role of different types of cells involved in regulations of the bridge among obesity, immunity, and systemic inflammation.

Type of Cell	Author and Year	Role of Cells	Interleukin Regulation Secretions	Chemoattractant Secretions
Macrophages	Huh et al., 2014 [25], Vieira-Potter et al., 2014 [26] Lumeng et al., 2013 [30] Mantovani et al., 2004 [31]	maintain the tissue homeostasis in normal weight protective and damaging functions in the metabolic regulations process	Macrophages exposed to immune complexes (IC) and LPS are characterized by an IL-10 high and IL-12 low phenotype and promote type II responses;	-
Neutrophils	Huh et al., 2014 [25], Vieira-Potter et al., 2014 [26] Bozkuș et al., 2018 [44], Atmaca et al., 2014 [45] Gordy et al., 2011 [65] Mărginean et al., 2019 [6] Dixon et al., 2006 [66] Asghar et al., 2017 [73]	maintain the tissue homeostasis in normal weight indicator of the inflammatory status being directly related to obesity degree connectors between innate and adaptive immunity components increase with obesity prime effectors of inflammatory responses	TNF α and IL-1β are secreted by neutrophils	-
Mast cells	Huh et al., 2014 [25], Vieira-Potter et al., 2014 [26]	maintain the tissue homeostasis in normal weight	-	-
Eosinophils	Huh et al., 2014 [25], Vieira-Potter et al., 2014 [26]	maintain the tissue homeostasis in normal weight	-	-
Lymphocytes	Bozkuș et al., 2018 [44], Atmaca et al., 2014 [45]	nutritional status and general stress	-	-
T Lymphocyte	Huh et al., 2014 [25], Vieira-Potter et al., 2014 [26] Mantovani et al., 2004 [31], Shaul et al., 2010 [32], Wentworth et al., 2010 [33] Patel et al., 2013 [28], Feuerer et al., 2009 [48]	maintain the tissue homeostasis in normal weight Th1 (M1) & Th2 (M2) lymphocyte polarizing signals, explanations for the differential responses to macrophages, ↑M1 gene markers & ↓M2 ones in adipose tissue	Th1—produces pro-inflammatory cytokines Th2—produces anti-inflammatory cytokinesTreg-↑ the adipokine from the visceral abdominal fat in obese mice	Regulatory T cell— inhibit macrophage migration and induce M2 polarization
B Lymphocyte	Huh et al., 2014, Vieira-Potter et al., 2014	maintain the tissue homeostasis in normal weight	-	-
Mast cells	Liu et al., 2009 [39]	a genetically induced deficiency or a pharmacological stabilization produced ↓ body weight gain	↓ adipokine levels	-
Eosinophils	Wu et al., 2009 [41]	a genetically induced eosinophils deficiency-↑ body weight, insulin resistance and impairment of glucose tolerance	-	-

Legend: IC—immune complexes, IL—interleukin, Th—T-helper, TNF–tumor necrosis factor, Treg—regulatory T cells.

**Table 2:** The role of cytokines produced by adipose tissue.

Type of Cytokines	Author and Year	Role of Cytokines	Observations
TNF	McGillicuddy et al., 2011 [74] Li et al., 2010 [36]	relationship between obesity-associated inflammation and insulin resistance	pro-inflammatory cytokines
Interferon	McGillicuddy et al., 2011 [74] Li et al., 2010 [36]	relationship between obesity-associated inflammation and insulin resistance	pro-inflammatory cytokines
IL-1	McGillicuddy et al., 2011 [74] Li et al., 2010 [36]	relationship between obesity-associated inflammation and insulin resistance	pro-inflammatory cytokines
IL-6	Donath et al., 2011 [92] Tilg et al., 2010 [93] Mărginean et al., 2020 [7]	precede the onset of type 2 DM involved in the pathogenesis of NAFLD	correlate with BMI in children with obesity central mediators of inflammatory reactions
IL-1β	Donath et al., 2011 [92] Tilg et al., 2010 [93]	preced the onset of type 2 DM involved in the pathogenesis of NAFLD	central mediators of inflammatory reactions
TNF α	Mărginean et al., 2020 [7]		correlate with BMI in children with obesity central mediators of inflammatory reactions

Legend: BMI–body mass index, DM–diabetes mellites, IL—interleukin, Th—T-helper, TNF–tumor necrosis factor, NAFLD–nonalcoholic fatty liver disease.
